# Schistosomes in the Persian Gulf: novel molecular data, host associations, and life-cycle elucidations

**DOI:** 10.1038/s41598-022-17771-2

**Published:** 2022-08-05

**Authors:** Maral Khosravi, David W. Thieltges, Jebreil Shamseddin, Simona Georgieva

**Affiliations:** 1 Department of Marine Ecology, GEOMAR Helmholtz Centre for Ocean Research Kiel, Düsternbrooker Weg 20, 24105 Kiel, Germany; 2 Department of Coastal Systems, NIOZ Royal Netherlands Institute for Sea Research, P.O. Box 59, 1790, AB Den Burg Texel, The Netherlands; 3 Infectious and Tropical Diseases Research CenterHormozgan Health Institute, Hormozgan University of Medical Sciences, Bandar Abbas, Iran; 4 Institute of Biodiversity and Ecosystem Research, Bulgarian Academy of Sciences, 2 Gagarin Street, 1113 Sofia, Bulgaria; 5 Department of Parasitology, School of Medicine, Chungbuk National University, Chungdae-ro 1, Seowon-gu, 28644 Cheongju, South Korea

**Keywords:** Biodiversity, Ecological epidemiology, Sequencing

## Abstract

Avian schistosomes, comprise a diverse and widespread group of trematodes known for their surprising ability to switch into new hosts and habitats. Despite the considerable research attention on avian schistosomes as causatives of the human cercarial dermatitis, less it is known about the diversity, geographical range and host associations of the marine representatives. Our molecular analyses inferred from *cox*1 and 28S DNA sequence data revealed presence of two schistosome species, *Ornithobilharzia canaliculata* (Rudolphi, 1819) Odhner, 1912 and a putative new species of *Austrobilharzia* Johnston, 1917. Molecular elucidation of the life-cycle of *O*. *canaliculata* was achieved for the first time via matching novel and published sequence data from adult and larval stages. This is the first record of *Ornithobilharzia* from the Persian Gulf and globally the first record of this genus in a potamidid snail host. Our study provides: (i) new host and distribution records for major etiological agents of cercarial dermatitis and contributes important information on host-parasite relationships; (ii) highlights the importance of the molecular systematics in the assessment of schistosome diversity; and (iii) calls for further surveys to reach a better understanding of the schistosome diversity and patterns of relationships among them, host associations, transmission strategies and distribution coverage.

## Introduction

Avian schistosomes comprise a diverse and widespread group known for their surprising ability to switch into new hosts and habitats^1^. Their cercariae are recognised as important causative agents of the waterborne allergic disease cercarial dermatitis (^2^ and references therein). However, the current systematics and taxonomy of the group is exclusively based on morphological characters of the adults. Difficulties in the identification of their larval stages and the lack of suitability of experimental approaches in large-scale screening studies of natural infections in intermediate hosts, has hindered the real assessment of their diversity, host and distributional ranges^3^. Often larval and adult stages from natural infections in snails and birds have been assigned to belong to the same species with the lack of further evidence linking their conspecificity. The discovery of avian schistosome diversity, their life-cycle elucidations and taxonomy has largely benefited from molecular phylogenetics studies (^2,4^ and references therein). To date, a total of 13 genera of avian schistosomes with about 70 species and 20 species-level genetically distinct lineages are known around the globe^4,5^. Based on the habitat where their life-cycles take place, avian schistosomes consist of freshwater and marine representatives.

Marine schistosomes represent a small group of widely distributed digeneans that are parasitic as adults in the vascular systems of various birds^6^. A predominant part of the extant marine schistosomes is known to parasitise charadriiforms (gulls and/or terns) with a few records in spheniscids^5,6^. Currently, four genera, *Austrobilharzia* Johnston, 1917, *Gigantobilharzia* Odhner, 1910, *Marinabilharzia* Lorenti, Brant, Gilardoni, Díaz & Cremonte, 2022 and *Ornithobilharzia* Odhner, 1912 are known to have marine-based life-cycles^4,5^. Of these, *Ornithobilharzia* Odhner, 1912 and *Austrobilharzia* Johnston, 1917 were recognised as an earlier diverging group which gave rise to all existent schistosomes^7^. Although, schistosomes represent a well-circumscribed monophyletic group, monophyly for the avian representatives has been rejected^4,7^. Phylogenetic hypotheses, revealed a basal switch from marine to freshwater environment which has occurred along a switch from caenogastropod to heterobranch snails^1^. A secondary switch from freshwater to marine environments has been suggested to have occurred with colonisation of heterobranch snails from the families Haminoeidae Pilsbry, 1895 and Siphonariidae Gray, 1827^8–10^.

*Ornithobilharzia canaliculata* was first described by Rudolphi (1819) as *Distoma canaliculatum*, the first schistosome species reported from the intestine of terns (“Sternae species brasilianae”) in Brazil^11^. In 1912, Odhner^12^ erected the genus *Ornithobilharzia* and defined *D. canaliculatum* as the type-species. Despite the wide range of known definitive hosts including marine birds of six genera (*Larus* L., *Sterna* L., *Chlidonias* Rafinesque, *Hydroprogne* Kaup, *Puffinus* (Manxsherwater), and *Thalasseus* F. Boie), and a wide geographical range across the Holarctic and Neotropics^13^, only a single marine gastropod species, *Lampanella minima* (Gmelin), has been assigned as the intermediate host in the Gulf of Mexico^14^. However, experimental elucidation of the life-cycle has never been carried out and a formal description of the cercaria of *O*. *canaliculata* is still lacking. Under the current taxonomic treatment, the genus includes three species: *O*. *amplitesta* Gubanov & Mamaev in Mamaev, 1959; *O. canaliculata* (Rudolphi, 1819) Odhner, 1912; and *O*. *lari* McLeod, 1937.

The closely-related genus *Austrobilharzia* Johnston, 1917 currently comprises 4 species: *A. odhneri* (Faust, 1924) Farely, 1971; *A. penneri* Short & Holliman, 1961; *A. terrigalensis* Johnston, 1917; and *A. variglandis* (Miller & Northup, 1926) Penner 1953. The genus was erected by Johnston (1917) to accommodate *A. terrigalensis*, a species found in the intestine of *Larus novae-hollandiae* shot at Terrigal, New South Wales, Australia. Caenogastropod snails have been reported as the natural intermediate hosts^15–18^. However, a combination of identification and taxonomic problems, have led to the biological paradox of a single species, *A*. *terrigalensis*, occurring at three distinct geographical regions and utilising different species of caenogastropod and bird hosts. Based on the geographical distribution, *A. terrigalensis* was assumed to occur in *Larus novae-hollandiae* and *Batillaria australis* in Australia; *A*. *valisineria*, *Mergus serrator* L., *Aythya affinis* Eyton, 1838 and *Ilyanassa obsoleta* (Say) in North America; and *Arenaria interpres* (L.) and *Littorina pintado* (W. Wood, 1828) in the Pacific.

Caenogastropods are one of the most diverse groups of gastropods comprising about 60% of the known species with predominantly marine forms^19^ and are known as intermediate hosts for a variety of trematode parasites^15,20,21^. Members of the genus *Pirenella* J. E. Gray are abundant inhabitants of intertidal sedimentary shores with wide geographical distribution ranging from the western Pacific and Indian Ocean to the eastern Mediterranean Sea. A recent study reported a total of 16 valid species within the genus, with some species known as inhabitants of extreme environments, from brackish estuaries to hypersaline lagoons and inland lakes^22^. *Pirenella cingulata* (Gmelin, 1791) is the most abundant caenogastropod species in the Persian and Oman Gulfs. It is known for its tolerance to environmental extremes and ability to flourish in intertidal muddy or sandy substrates, as well as mudflats adjacent to mangrove forests^23,24^.

As part of an ongoing study aiming to characterise trematode diversity in the horn snail (*Pirenella cingulata*) along the coast of Iran, we here report on the diversity of avian schistosomes associated with marine life-cycles using *cox*1 and 28S rDNA sequence data. The present study is the first to molecularly elucidate the life-cycle of the first ever described schistosome, *O*. *canaliculata*, and further reports on a putative new species of *Austrobilharzia.* Both species recovered are of the largely understudied marine schistosomes known for their implication as causative agents of cercarial dermatitis. This is the first unambiguous documentation that the potamidid snail *P*. *cingulata* is the natural snail host for *O*. *canaliculata*. The evolutionary relationships and host-parasite associations among the avian schistosomes are further revisited.

## Results

**Figure 1:**
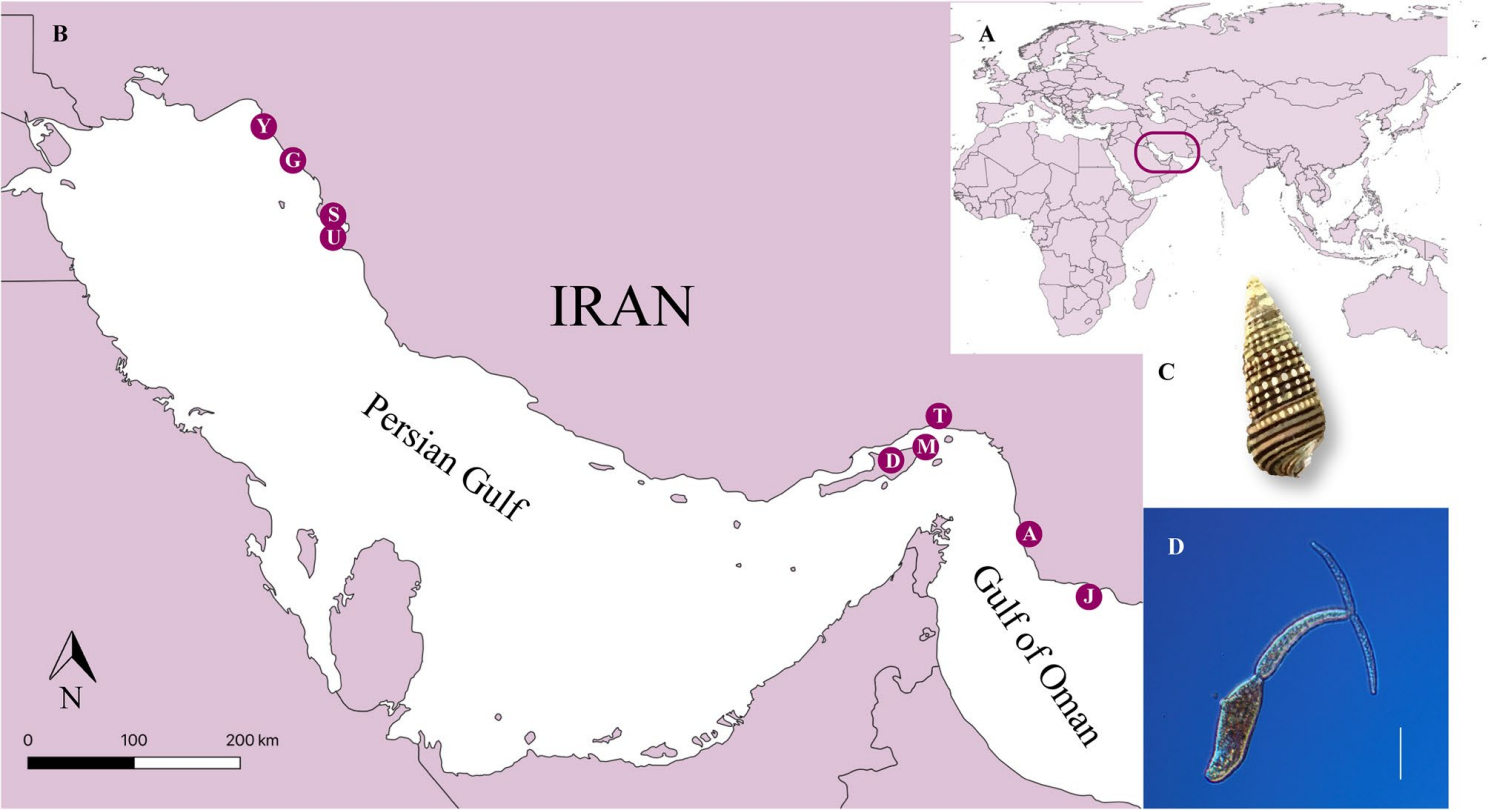
(**A**) General view map, generated using QGIS version 3.4 (http://www.qgis.org)^52^, and (**B**) sampling localities along the Persian Gulf and the Gulf of Oman off Iran. Points correspond to the sampling localities. *Abbreviations*: A, Azini; D, Dargahan; G, Genaveh; J, Jask; M, Geshm; S, Shif; T, Bandar Abbas; Y, Deylam; U, Bushehr. (**C**) Snail intermediate host *Pirenella cingulata* (Gmelin, 1791). (**D**) Cercaria collected from *P*. *cingulata*. *Scale-bar* = 100 µm.

Three out of the 1,745 examined *P*. *cingulata* were infected with avian schistosomes. The infected snails were collected at two distinct localities named Genaveh (*n* = 2; *prevalence* = 1%) and Jask (*n* = 1; *prevalence* = 0.4%) (see also Fig. 1 for sampling locations). Successful amplifications were achieved for 28S and *cox*1 for all three isolates. The yielded sequences were 1254–1285 bp (28S rDNA) and 344–730 bp long (*cox*1). The two isolates from Genaveh shared an identical 28S rDNA sequence with a published isolates for *Ornithobilharzia canaliculata* from the USA ex *Larus delawarensis* and *L*. *occidentalis* (AF167085, AY157248, KP734309), while the isolate from Jask differed by 2.3% (29 bp) from the former ones. A BLASTn search indicated that the latter isolate belonged to the genus *Austrobilharzia*. The novel isolate from Iran differed by 12–19 bp (0.9–1.8%) from the published representatives of the genus. The closest relative was an otherwise unidentified isolate from the same host species, *P*. *cingulata*, from off Kuwait (12 bp, 0.9% genetic difference).

*Cox*1 sequence divergence between our two isolates of *O*. *canaliculata* from Iran was 9 bp (2.6%). In contrast to the identical 28S sequences between the novel and published isolates for *O*. *canaliculata*, *cox*1 sequences differed substantially, ranging between 27 and 32 bp (7.9–9.3%). The single isolate from Jask differed by 1.66–1.82% (49–55 bp; 16.3–18.2%) from the novel isolates for *O*. *canaliculata*, and by 59 bp (20.1%) from *Austrobilharizia* sp. from Kuwait. Interspecific sequence divergence within *Austrobilharzia* was within the range of 21–63 bp (9.7–20.1%). However, the intergeneric divergence between the isolates for *Ornithobilharzia* and *Austrobilharzia* was somehow lower that the interspecific divergence for *Austrobilharzia*, i.e., 25–61 bp (7.7–18.4%). A single *cox*1 isolate for *Austrobilharzia variglandis* ex *Larus* sp. from Canada was not included in the sequence comparisons as it covers a distinct region of the *cox*1 gene and did not align with the remaining published isolates.

**Figure 2:**
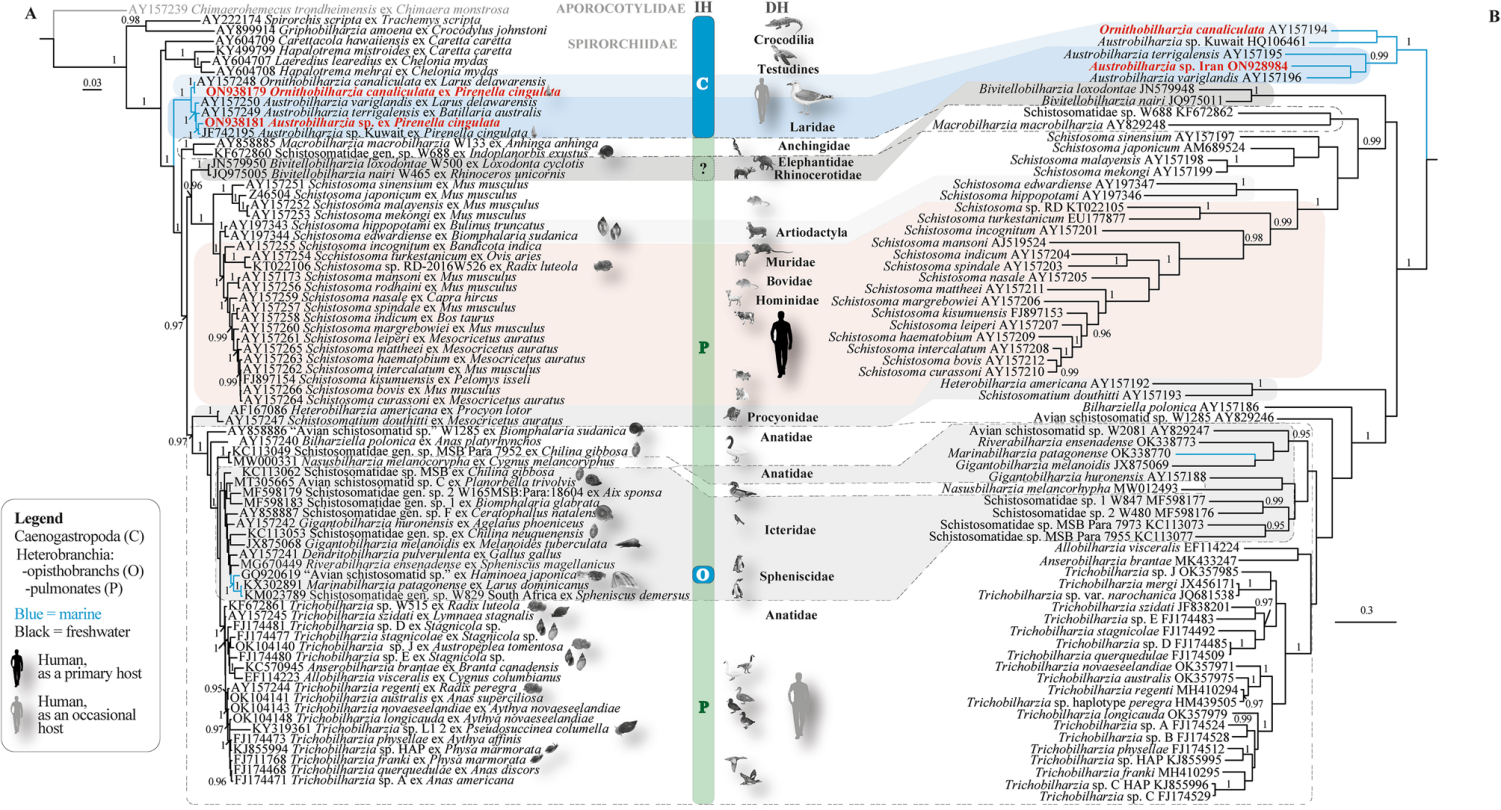
Bayesian analyses of the (**A**) 28S rDNA and (**B**) *cox*1 datasets constructed using MrBayes v. 3.2.3 under the GTR + I + Г model of sequence evolution. Analyses were run for 10,000,000 generation and 25% discarded as "burn-in". Posterior probability values are given above the branches; values. Nodes with < 0.95 posterior probability support have been collapsed. Branch length scale-bar indicates number of substitutions per site. Newly-generated sequences are indicated in colour indicated red and bold. Hosts of origin of individual sequences are indicated after the specimen’s host name. Branches in blue indicate schistosomes with marine life-cycle. Shaded areas and taxa outlined with doted lines reflect on the current taxonomic framework of the family and also given on the right.

The aligned 28S dataset consisted of 76 terminals (2 newly-sequenced) and it was 1370 bp long, 78 of which were excluded prior to analyses. The *cox*1 dataset comprised 66 terminals and it was 1031 bp long. Analyses of the individual genes resulted in well-resolved trees (Fig. 2). The 28S rDNA hypothesis, presented in Fig. 2A, included representatives of all named and molecularly characterised species-level lineages except for the monotypic *Jilinobilharzia* as molecular data currently do not exist (the single species, *J*. *crecci* Liu & Bai, 1976, has not been reported since its original description). Therefore, the ingroup taxa consisted of representative sequences of the families Schistosomatidae and the closely related Spirorchiidae (see Supplementary Table S1). The outgroup comprised representative of the Aporocotylidae and it was informed from previous phylogenies^25^. Our phylogenetic hypothesis recovered the spirorchiids in freshwater crocodilian and testudine hosts as the earliest diverging lineage. Spirorchiids with marine life-cycle clustered in a distinct clade basal to the Schistosomatidae. Members parasitic in marine testudines were identified as a distinct clade sister to all remaining schistosomes parasitic in birds and mammals. Schistosomes clustered into four distinct lineages: (i) an earlier diverging and strongly supported clade comprising the marine *Ornithobilharzia* and *Austrobilharzia* (ii) *Macrobilharzia*—a genus known from suliform birds which was resolved as a distinct lineage basal to the freshwater schistosomes, and two strongly-supported multi-taxa sister clades predominantly of (iii) mammalian and (iv) avian schistosomes. The mammalian schistosomes were further recovered as three distinct lineages: (i) *Bivitelobilharzia*—a genus including species parasitic in elephants and rhinoceros were recovered in a strongly-supported sub-clade sister to the main clade of mammalian schistosomes; (ii) a sub-clade of *Schistosoma* spp. with South East Asian distribution; and (iii) a clade comprising African representatives of *Schistosoma*. The North American mammalian representatives, *Heterobilharzia* and *Schistosomatium* were resolved as closer relatives to the large clade of avian schistosomes (*Trichobilharzia* + *Marinabilharzia* + *Dendritobilharzia* + *Gigantobilharzia* + *Nasusbilharzia* + *Riverabilharzia*). The remaining avian schistosomes clustered in two sister monophyletic clades with generally strong support for the major nodes. *Bilharziella* and *Nasusbilharzia* were recovered as earlier diverging to the two sister strongly-supported subclades of *Gigantobilharzia* + *Dendritobilharzia* + *Marinabilharzia* + *Riverabilharzia*, and *Trichobilharzia* + *Allobilharzia* + *Anserobilharzia*.

The *cox*1 tree was well-resolved and received strong support for most of the internal nodes (Fig. 2B). Taxa largely grouped in consistence with the 28S solution. *Ornithobilharzia* and *Austrobbilharzia* clustered into two distinct strongly-supported sister clades. The newly-sequenced isolate from Jask clustered in a clade with *A. variglandis* and *A. terrigalensis*; however, the isolate for *O*. *canaliculata* clustered with otherwise unidentified isolate labelled as *Austrobilharzia* sp. from Kuwait indicating a possible misidentification of the latter one. This was further confirmed by the high levels of genetic divergence in comparison with the other isolates of *Austrobilharzia* as indicated above.

## Discussion

The present study is part of an effort to document the trematode diversity in *P. cingulata* (Gmelin, 1791), one of the most abundant snail species along the Iranian coast^23,24,26^. Sequence data for two species of marine avian schistosomes, *Ornithobilharzia canaliculata* (Rudolphi, 1819) and a putative new species of *Austrobilharzia* Johnston, 1917, are represented in a phylogenetic context together with other members of the family Schistosomatidae. This is the first report and molecular evidence for *Ornithobilharzia canaliculata* (Rudolphi, 1819) infecting *P. cingulata* as an intermediate host and it is the first partial molecular elucidation of its life-cycle. Our study adds to the diversity, host associations and phylogeny of the avian schistosomes with marine-based life-cycles, a group of schistosomes with great etiological importance.

Cercariae of the marine schistosomes are recognised as important etiological agents of human dermatitis^27–29^. Despite their importance to the public health, still very little is known about their diversity and evolution^7^ as a consequence of largely under surveyed marine habitats for schistosomes worldwide^4^. This is in sharp contrast with the wealth of knowledge gathered about the mammalian and avian schistosomes with freshwater-based life-cycles, and information concerning the natural history of most marine schistosomes is scarce. The slow rates in recovering marine schistosomes, low species richness recorded in snail hosts and the convoluted taxonomy of the group, including separate taxonomic treatments of the distinct life-cycle stages, reflects the scarcity of data^30^. Matching sequence data for different life-cycle stages and across distant localities has accelerated life-cycles elucidations and host-parasite associations^4,5^. Although, the molecular systematics has had a major impact for the recent increase in discoveries and species delimitation, it has led to a plethora of putative new species and lineages of avian schistosomes for which only molecular data for their cercarial stages exist. Most of these putative species/species level lineages are of considerable importance due to their etiological significance. Their formal descriptions await as reliably identified adult stages are needed to help infer on their respective life- cycles and host-parasite associations.

*Ornithobilharizia canalicata* was originally described from *Sterna galericulata* in Brazil^11^. Later the species was reported from a wide range of gulls and terns across the Americas, Europe, Asia and New Zealand (see Table 1 for details). *Larus dominicanus* Lichtenstein and *L. maculipennis* Lichtenstein serve as the main hosts in the southern hemisphere; *Larus delawarensis* Ord, and *L*. *occidentalis* Audubon have been reported as hosts in North America and a total of 22 species of gulls and terns were reported as hosts across Europe and the Middle East. Despite the large number of definitive hosts, thus far the species was reported only from a single mollusc species, *Lampanella minima* (Gmelin), in North America. However, an experimental infection linking larval and adult stages has never been conducted. An important result from our study is the molecular confirmation of the conspecificity of our isolate from *P*. *cingulata* with the published isolate of an adult worm from North America. Matching sequence data for isolates from different life-cycle stages collected from disparate locations and times, provides unambiguous link between adult and larval stages from natural infections and accelerates species circumscription. The intercontinental distribution and the rather narrowly defined clade of gulls is instructive for studies on the transmission of avian zoonoses and the epidemiology of human cercarial dermatitis. The trans-continental distribution of *Ornithobilharzia* across the America, Europe and Asia is an explicit example that species dispersal is determined by the most vagile, bird host, involved in the trematode life-cycle. It is widely accepted that the distribution of the definitive host governs the larval trematode recruitment in the snail (first) intermediate host (^31^ and references therein). Resolving the relative roles of both host ecology and phylogeny in respect to the parasite transmission dynamics over evolutionary times would require further concerted efforts. Phylogenetic studies based on denser and wide taxon sampling including diverse intermediate and definitive hosts is crucial for building up an improved framework and better interpretation of the schistosome biology^3^. Further, good documentation and re-evaluation of the morphological charters of the respective larval stages is urgently needed.

**Table 1:** Records of *Ornithobilharzia* spp*.* and *Austrobilharzia* spp.

Species	Host	Locality	References
*Austrobilharzia terrigalensis* Johnston, 1917	*Batillaria australis* (Quoy & Gaimard)	Australia: Iron Cove, Sedney Harbour	Lockyer et al.^68^
	*Batillaria australis* (Quoy & Gaimard)	Australia	Walker^69^
	*Batillaria australis* (Quoy & Gaimard)	Australia: Swan Estuary	Appleton^70^
	*Batillaria australis* (Quoy & Gaimard)	Australia: Swan Estuary	Appleton^71^
	*Batillaria australis* (Quoy & Gaimard)	Australia	Johnston^72^ sensu Farley^13^
	*Batillaria australis* (Quoy & Gaimard)	Australia	Appleton^73^
	*Batillaria australis* (Quoy & Gaimard)	Australia: Narrabeen Lagoon	Bearup^74^
	*Cerithideopsis scalariformis* (Say)	North America	Holliman^17^
	*Cerithideopsis scalariformis* (Say)	North America	Short and Holliman^18^
	*Ilyanassa obsoleta* (Say)	USA	Miller and Northup^36^ sensu Farely^13^
	*Ilyanassa obsoleta* (Say)	USA	Camishion et al.^75^
	*Ilyanassa obsoleta* (Say)	USA: New Jersey	Zibulewsky et al.^76^
	*Ilyanassa obsoleta* (Say)	USA: Atlantic coast	Bacha et al.^77^
	*Ilyanassa obsoleta* (Say)	USA: Atlantic coast	Wood and Bacha^78^
	*Ilyanassa obsoleta* (Say)	USA: North Carolina	Sindermann^79^
	*Ilyanassa obsoleta* (Say)	USA: California	Grodhaus and Keh^28^
	*Littorina pintado* (W. Wood)	US: Hawaii	George et al.^80^
	*Littorina pintado* (W. Wood)	Hawaii	Chu and Cutress^37^ sensu Farely^13^
	*Planaxis sulcatus* (Born)	Australia: GBR, Heron Island	Rohde^81^
	*Planaxis sulcatus* (Born)	Australia	Rohde^81^
	*Anous minutus* (L.)	Australia: GBR, Heron Island	Rohde^81^
	*Arenaria interpres* (L.)	Hawaii	Chu and Cutress^37^ sensu Farely^13^
	*Arenaria interpres* (L.)	US: Hawaii	George et al. ^80^
	*Aythya affinis* (Eyton)	USA: Massatchusetts	Price^82^ sensu Farely^13^
	*Aythya valisineria* (Wilson)	Canada	McLeod^83^ sensu Farley^13^
	*Larus californicus* Lawrence	USA: Wyoming	Keppner^84^
	*Larus novaehollandiae* Stephens	Australia	Johnston^85^ sensu Farley^13^
	*Larus novaehollandiae* Stephens	Australia: Swan Estuary	Appleton^86^
	*Larus novaehollandiae* Stephens	Australia: Swan Estuary	Appleton^73^
	*Mergus serrator* L	USA	Penner^87^ sensu Farley^13^
	*Egretta sacra* (Gmelin)	Australia	Rohde^81^
	*Egretta sacra* (Gmelin)	Australia: GBR, Heron Island	Rohde^81^
	*Larus novaehollandiae* Stephens	Australia	Johnston^85,88^, Appleton^73^
	*Larus novaehollandiae* Stephens	Australia: Terrigal, near Sydney	Johnston^85^
	*Larus novaehollandiae* Stephens	Australia: GBR, Heron Island	Rohde^81^
	*Larus novaehollandiae* Stephens	Australia	Rohde^81^
	*Larus hemprichii* Bruch	Red Sea	Witenberg and Lengy^89^
	*“Canary”* (exp.)	USA: California	Grodhaus and Keh^28^
*Austrobilhariza variglandis* (Miller & Northup, 1926)	*Ilyanassa obsoleta* (Say)	USA: Delaware estuaries	Curtis^90^
	*Ilyanassa obsoleta* (Say)	USA: Delaware estuaries	Curtis and Tanner^91^
	*Ilyanassa obsoleta* (Say)	USA: Mumford Cove, Connecticut	Barber and Caira^92^
	*Ilyanassa obsoleta* (Say)	North America	Grodhaus and Keh^28^, Curtis^90^, Leighton et al.^93^
	*Ilyanassa obsoleta* (Say)	USA: Little Egg Inlet, New Jersey	Ferris and Bacha^94^
	*Littorina pintado* (W. Wood)	USA: Hawaii	Chu and Cutress^37^
	*Anous stolidus pileatus* (Scopoli)	USA: Hawaii	Chu and Cutress^37^
	*Arenaria interpres* (L.)	USA: Hawaii	Chu and Cutress^37^
	*Aythya affinis* (Eyton, 1838)	USA: Eastern part	Price^82^
	*Branta canadensis* (L.)	USA: Mumford Cove, Connecticut	Barber and Caira^92^
	*Larus argentatus* Pontoppidan	USA: Mumford Cove, Connecticut	Barber and Caira^92^
	*Larus argentatus* Pontoppidan (exp.)	USA	Stunkard and Hinchliffe^95,96^
	*Larus delawarensis* Ord	USA: Mumford Cove, Connecticut	Barber and Caira^92^
	*Larus delawarensis* Ord	USA: Delaware	Lockyer et al.^68^
	*Larus marinus* L	USA: Mumford Cove, Connecticut	Barber and Caira^92^
	*Larus marinus* L	North America	Keppner^84^, Barber and Caira^92^
	*Larus novaehollandiae* Stephens	Australa: Heron Island	Rohde^81^
	*Mergus serrator* L	USA	Penner^87^
	*Mergus serrator* L	North America: Hawaii	Penner^97^
	*Phalacrocorax auritus* (Lesson)	USA: Mumford Cove, Connecticut	Barber and Caira^92^
	*Phalacrocorax auritus* (Lesson)	North America	Barber and Caira^92^
	*Sterna fusccata oahuensis* (L.)	USA: Hawaii	Chu and Cutress^37^
*Austrobilharzia odhneri* (Faust, 1924) Farley, 1971	*Numenius arquata* (L.)	China	Faust^98^
*Austrobilharzia penneri* Short & Holliman, 1961	*Cerithideopsis scalariformis* (Say)	North America	Holliman^17^
	*Cerithideopsis scalariformis* (Say)	North America	Short and Holliman^18^
	*Cerithidea scalariformis* and “parakeets, chickens and pigeons (exp.)”	USA: Florida, Northern Gulf coast	Short and Holliman^18^
*Austrobilhariza* sp.	*Cerithideopsis californica* (Haldeman)	USA: Bolinas Lagoon, in central California	Sousa^99^
	*Cerithidia* sp.	North America	Martin^16^
	*Littorina pintado* Wood	North America: Hawaii	Chu^100^
	*Pirenella cingulata* (Gmelin)	Kuwait: Kuwait Bay	Al-Kandari et al.^15^
	*Pirenella cingulata* (Gmelin)	Kuwait Bay	Al-Kandari et al.^15^
	*Nassarius (Hinia) reticulatus* (L.)	Italy	Canestri-Trotti et al.^101^
	*Littorina keenae* Rosewater	North America	Penner^102^
	*Planaxis sulcatus* (Born)	Kuwait: Kuwait Bay	Abdul-Salam and Sreelatha^103^
	*Anous minutus* Boie	North America: Hawaii	Chu^37^
	*Gavia immer* (Brünnich)	North America	Kinsella and Forrester^104^
	*Larus dominicanus* Lichtenstein	South Africa	Appleton^105,106^
	*Larus dominicanus* Lichtenstein	South Africa: Umgeni Estuary	Appleton^105^
	*Onychoprion fuscatus* L	North America: Hawaii	Chu^37^
	*Pelecanus occidentalis* L	North America	Courtney and Forrester^107^
*Ornithobilharzia canaliculata* (Rudolphi, 1819)	*Lampanella minima* (Gmelin)	North America	Penner^14^, Morales et al.^108^
	*Lampanella minima* (Gmelin)	USA: Florida	Morales et al.^108^
	*Lampanella minima* (Gmelin)	Brazil	Travassos et al.^109^
	*Chlidonias hybrida* (Pallas)	Caspian Sea	Saidov^110^, Bykhovskaya^111^
	*Hydroprogne caspia* (Pallas)	Black Sea, Central Europe	Leonov^112^, Macko^113^
	*Hydroprogne caspia* (Pallas)	West Siberia	Bykhovskaya^114^
	*Ichthyaetus melanocephalus* Temminck	Calabria, Southern Italy	Santoro et al.^115^
	*Larus fuscus* L	Red Sea	Witenberg and Lengy^89^
	*Larus argentatus* Pontoppidan	West Siberia	Bykhovskaya^114^
	*Larus cachinnans* Pallas	Spain, Galicia	Sanmartín et al.^116^
	*Larus canus* L	Black Sea	Popova^117^, Bykhovskaya^111^
	*Larus delawarensis* Ord	Canada	McLeod^83^
	*Larus delawarensis* Ord	USA: Donley County, Texas	Lockyer et al.^68^
	*Larus delawarensis* Ord	USA: Texas	Snyder and Locker^7^
	*Larus dominicanus* Lichtenstein	Brazil	Travassos^118^
	*Larus dominicanus* Lichtenstein	New Zealand	Rind^119^
	*Larus dominicanus* Lichtenstein	Argentina	Szidat^120^
	*Larus maculipennis* Lichtenstein	Argentina	Szidat^120^
	*Larus fuscus* L	North Russia, Red Sea	Shygin^120^, Bykhovskaya^111^, Witenberg and Lengy^89^
	*Larus fuscus* L	Sweden	Odhner^12^
	*Larus hemprichii* Bruch	Red Sea	Witenberg and Lengy^89^
	*Larus ichthyaetus* Pallas	Black Sea	Leonov^112^
	*Hydrocoloeus melanocephalus* (Temminck	Italy	Parona and Ariola^121^
	*Hydrocoloeus minutus* (Pallas)	Caspian Sea	Saidov^110^, Bykhovskaya^111^
	*Larus occidentalis* Audubon	USA	Jothikumar et al.^122^
	*Larus ridibundus* L	North Russia, Caspian Sea	Shigin^123^, Saidov^110^, Bykhovskaya^111^
	*Larus ridibundus* L	West Siberia	Bykhovskaya^114^
	*Puffinus kuhli* (Boie)	Red Sea	Witennberg^124^
	*Sterna galericulata*	Brazil	Rudolphi^11^
	*Sterna hirundo* L	Czech Republic	Kolářová et al.^51^
	*Sterna sandwichensis* Latham	Black Sea	Leonov^112^
*Ornithobilharzia* sp. (?*canaliculata*)	*Eudocimus albus* (L.)	North America	Bush and Forrester^125^
*Ornithobilharzia lari* McLeod, 1937	*Larus argentatus* Pontoppidan	Canada. Nova Scotia	McLeod^83^
	*Larus delawarensis* Ord	Canada. Nova Scotia	McLeod^83^
	*Larus philadelphia* (Ord)	Canada. Nova Scotia	McLeod^83^
*Ornithobilharzia amplitesta* Gubanov & Mamaev in Mamaev, 1959	*Tringa glareola* L	Russia	Mamaev^126^

Successful transmission of parasites with complex life-cycles requires an overlap of all hosts involved. The invertebrate first intermediate host has been recognised as one of the keys to the evolutionary expansions of the digenean trematodes. All schistosomes (marine and freshwater) are known to develop in gastropods. The basal position of the marine schistosomes (*Austrobilharzia* and *Ornithobilharzia*) has been considered as an indication for a successful ancestral marine-transmitted bird parasite transmission in colonising both freshwater snails and mammals^25^. The schistosomes emerging from marine heterobranch snails (*Haminea* and *Siphonaria*) and also recorded in penguins are a well-known example of secondary colonisation of marine habitats by the schistosomes^30,32,33^. Considering the snail intermediate hosts, in at least two instances, even congeneric schistosomes depend on markedly divergent gastropod lineages, i.e., pulmonates *versus* opisthobranchs or caenogastropods, indicative for an extensive host switching within the molluscan hosts^34^ and references therein).

Avian schistosomes are known to have colonized a wide range of snail hosts with representatives from 15 snail families: (i) caenogastropods from both marine (Potamididae, Batilariidae, Nassariidae, and Littorinidae^14,15,35–37^ and freshwater environments (Thiaridae, Ampullariidae, Hydrobiidae, and Semisulcospiridae^35,38–41^; (ii) heterobranchs from marine (Haminoeidae^8,9^, and freshwater (Valvatidae^42^; and (iii) pulmonates from marine (Siphonariidae^10^) and freshwater (Physidae, Lymnnaeidae, Planorbidae, and Chilinidae^1,3,35,43,44^. Reports of avian schistosomes from distantly related snail intermediate hosts are not rare and invoke questions on the proper identification of the respective parasites. *Dendritobilharzia pulverulenta*^45^ Skrjabin, 1924 has been reported from two distinct planorbid snails *Gyraulus* Charpentier, 1837 and *Anisus vortex* (L.)^46^. *Gyraulus* has been reported as a natural host of the species in North America and New Zealand, while *Anisus* and *Planorbis planorbis* (L.) have been reported as hosts in Europe. *Trichobilharzia jequitibaensis* Leite, Costa, & Costa, 1978 is known to infect both lymnaeid and physid snails^47^. *Austrobilharzia terrigalensis* has been considered to utilise distinct snail hosts across its distributional range in Australia (*Batillaria australis* (Quoy & Gaimard)), North America (*Cerithideopsis scalariformis* (Say), and *Ilyanassa obsoleta* (Say) and the Pacific (*Littorina pintado* (W. Wood)). Intercontinental and trans-hemispheric distribution has been recently reported for *Trichobilharzia querquedule*^48,49^, however the species is known as a parasite specific to *Physa* spp. as an intermediate host.

In respect to their definitive hosts, a predominant part of the schistosomes is known as parasitises in birds. Currently a total of 13 genera are known as parasites in birds: *Austrobilharzia* Johnston, 1917 (6 species), *Allobilharzia* (1 species), *Anserobilharzia* (1 species), *Bilharziella* (1 species), *Dendritobilharzia* (2 species) *Gigantobilharzia* (*c*.14 species), *Jilinobilharzia* (1 species), *Macrobilharzia* Travassos, 1922 (2 species), *Ornithobilharzia* Odhner, 1912 (3 species); *Nasusbilharzia* Flores^50^ (1 species), *Marinabilharzia* (1 species), *Riverabilharzia* (1 species)^5^ and *Trihobilharzia* (*c*.35 species). Among them, species of *Trihobilharzia* have been subject of the most intensive research due to their recognition as leading etiological agents of human cercarial dermatitis (^51^ and references therein). The genus represents the most speciose among the bird schistosomes with about 35 species or species level lineages. However, about 65% of the remaining avian schistosomes, yet remain largely unstudied with fragmentary data on their diversity, biology and ecology. This is especially true in respect to the marine schistosomes for which life-cycle information lags behind their freshwater relatives. Despite the great efforts made so far in building up a comprehensive framework for the study of schistosome diversity, still considerable data are needed for assessing their true diversity due to the difficulty of directly relating larval and adult stages. Consistent efforts towards the use of integrative approach including collecting novel data from diverse host species and combining them thorough morphological examination, traditional systematics, classical taxonomy and phylogenetics have been proven as most valuable practice providing important information to the better understanding of the biodiversity and evolutionary relationships of the group^2–4^.

Our study strongly suggests that the biodiversity of the marine schistosomes is underestimated. The extensive discovery-based studies about schistosome diversity during the last two decades has revealed an immense diversity of avian schistosomes. However, unravelling their true diversity across hosts and geographic areas have been hindered by the difficulties of matching distinct life-cycle stages. There is still a need for more records of identifiable adult and larval schistosomes. Our study is a crucial step towards better understanding of important properties of marine schistosome biology and ecology, their patterns of diversification and distribution.

## Materials and methods

### Host and parasite collection

A total of 1745 adult *P*. *cingulata* (Gmelin, 1791) were sampled from 9 distinct locations along the Iranian coastline between December 2019 and February 2020 (Fig. 1). Samples comprised a minimum of 200 individual snails per locality, which were opportunistically collected by hand at the low tide from the intertidal zone. Snails were transferred alive to the laboratory, where they were measured (length and weigh) and labelled with a unique code given to each specimen. Each snail was then placed in an individual 50 ml beaker filled with filtered seawater and exposed to a warm light source for 3–4 h to simulate cercarial emergence. Beakers were screened under a stereomicroscope for the presence of cercariae indicating patent infections in the snail host. Prepatent infections were detected with snail dissections, which were conducted on the 3rd day of light stimulation. Both the released cercariae and schistosome sporocysts recovered from the host’s tissue were washed with distilled water and preserved in molecular grade ethanol for DNA isolation and sequencing.

### Sequence generation

Ethanol-preserved samples of pooled cercariae were subjected to DNA extraction and sequencing. Partial *cox*1 and 28S rDNA sequences were generated for the schistosome parasites recovered in order to achieve molecular identification and carry out reconstruction of their evolutionary relationships using published primers (28S (digl2 + 1500R^53,54^; ECD2 + 900F^55,56^ as internal sequencing primers; *cox*1: JB3 + JB4.5^57^ or CO1-R^58^). Contiguous sequences were aligned with MAFFT v.7^59,60^ as an online execution. After alignment, sequences for *cox*1 were checked for stop codons using the echinoderm and flatworm mitochondrial code (translation table 9^61^). All sequences were trimmed in order the first base to correspond to the first codon position in order to simplify position-coding in the downstream analyses.

### Phylogenetic analyses

Phylogenetic analyses were performed on individual gene datasets using Bayesian inference (see Supplementary Table 1 for details on the taxa included in the analyses). Prior to analyses, the 'best-fitting' models of nucleotide substitution were estimated based on the Bayesian information criterion (BIC) in jModelTest v. 2.1.4^62^. BI analysis was carried out with MrBayes v. 3.2.7^63^ on the CIPRES Science Gateway v.3.3^64^ using Markov chain Monte Carlo (MCMC) searches on two simultaneous runs of four chains for 10^7^ generations, sampling trees every 10^3^ generations. The “burn-in” determined by stationarity of lnL assessed with Tracer v.1.5^65^ was set for the first 25% of the trees sampled, and a consensus topology and nodal support estimated as posterior probability values^66^ were calculated from the remaining trees. Phylogenetic trees were visualized and finalised in FigTree v. 1.4.4^67^. The newly-generated sequences were deposited in GenBank under accession numbers: ON928982–ON928984 (*cox*1), ON938179–ON938181 (28S) in the case of avian schistosomes, and ON911910 (*cox*1), ON911912 (28S)—for the snail host.

## Supplementary Information

Supplementary Information.

## Data Availability

All data are available in the main manuscript or additional supporting files.
